# Do agri-food market incentives improve food security and nutrition indicators? a microsimulation evaluation for Kenya

**DOI:** 10.1007/s12571-021-01215-2

**Published:** 2021-09-30

**Authors:** María Priscila Ramos, Estefanía Custodio, Sofía Jiménez, Alfredo J. Mainar-Causapé, Pierre Boulanger, Emanuele Ferrari

**Affiliations:** 1 Facultad de Ciencias Económicas, Departamento de Economía. CONICET-Universidad de Buenos Aires, Instituto Interdisciplinario de Economía Política de Buenos Aires (IIEP-Baires), Universidad de Buenos Aires, Avda. Córdoba 2122 C1120AAQ, Buenos Aires, Argentina; 2 Centre d’Etudes Prospectives et d’Information Internationale (CEPII), 20 avenue de Ségur, 10726 75334 Paris cedex 07, TSA France; 3 European Commission, Joint Research Centre (JRC), Edificio Expo, Calle Inca Garcilaso, 41092 Seville, Spain; 4 Instituto de Salud Carlos III, Centro Nacional de Medicina Tropical, Avda. Monforte de Lemos, 5, 28029 Madrid, Spain; 5 Departamento de Análisis Económico, Universidad de Zaragoza, Gran Vía 2, 50001 Zaragoza, Spain; 6 Departamento de Economía Aplicada III, Universidad de Sevilla, Avda. Ramón y Cajal, 1, 41018 Seville, Spain

**Keywords:** Food security, Nutrition, Household survey, Microsimulations, Market access, Kenya, Africa, C14, C83, I38, Q18

## Abstract

The sustainable development goal #2 aims at ending hunger and malnutrition by 2030. Given the numbers of food insecure and malnourished people on the rise, the heterogeneity of nutritional statuses and needs, and the even worse context of COVID-19 pandemic, this has become an urgent challenge for food-related policies. This paper provides a comprehensive microsimulation approach to evaluate economic policies on food access, sufficiency (energy) and adequacy (protein, fat, carbohydrate) at household level. The improvement in market access conditions in Kenya is simulated as an application case of this method, using original insights from households’ surveys and biochemical and nutritional information by food item. Simulation’s results suggest that improving market access increases food purchasing power overall the country, with a pro-poor impact in rural areas. The daily energy consumption per capita and macronutrients intakes per capita increase at the national level, being the households with at least one stunted child under 5 years old, and poor households living areas outside Mombasa and Nairobi, those which benefit the most. The developed method and its Kenya's application contribute to the discussion on how to evaluate nutrition-sensitive policies, and how to cover most households suffering food insecurity and nutrition deficiencies in any given country.

**Supplementary Information:**

The online version contains supplementary material available at 10.1007/s12571-021-01215-2.

## Introduction

The sustainable development goal #2 (SDG#2) or “zero hunger” aims at ending hunger and malnutrition by 2030. The challenge of ensuring access to safe, nutritious and sufficient food for all people all year round and eradicating all forms of malnutrition is increasingly complicated by the surge of the COVID-19 pandemic. Global estimates on food security and nutrition (FS&N) show that the global community is falling far short in this goal, with numbers of food insecure and malnourished people on the rise. Between 720 and 811 million people in the world faced hunger in 2020, and the global burden of malnutrition remained a challenge with 149 million of children stunted and 45 million wasted (FAO et al., 2021).

The five targets within the SDG#2 refer to food security, nutrition, and different dimensions of agriculture, assuming the enormous potential of agriculture for contributing to the end of hunger and malnutrition. Agriculture can promote FS&N outcomes, but the linkages are complex, and influenced by diverse factors such as the food production systems, the market structures and consumer demands, as well as nutrition relevant policies and programs (Blesh et al., 2019; Pandey et al., 2016). This complexity is a key reason explaining the challenge of measuring the impact of agricultural policies on FS&N (Qureshi et al., 2015) and the scarce evidence on the nutritional effect of agricultural interventions (Masset et al., 2012). In addition, key gaps hinder the provision of this sound evidence. They are related to: (i) the scarcity of data availability, referring to specialized household surveys collecting economic related variables as well as detailed food consumption and nutrition data, (ii) the need to use proper metrics, and (iii) the call to build more nutrition-sensitive simulation models to gauge the FS&N impacts of large-scale policies and programs.

Agricultural FS&N-sensitive policies should be simulated at the dimension of its real implementation (macro or sectoral levels), which is already well addressed by simulations models, but policies FS&N impacts should be measured at the individual or household levels with the properly data match, FS&N metrics and modelling of pathways (i.e., prices and quantities). Economic simulation models (Beyene et al., 2018; Boulanger et al., 2020; Deaton, 1989a, 1989b, 1997; Pauw et al., 2018) focus on poverty, welfare and food purchasing power as national or household agricultural policy impacts. On the other hand, medical microsimulation models target individuals and their households about the change in their nutrients intakes due to food polices to reduce health costs or diseases (Basu et al., 2013, 2018; Chi et al., 2014; Mozaffarian et al., 2018). In this sense, each discipline tells the FS&N story from only one dimension of the problem. To measure the impact of policies on FS&N in a comprehensive manner, these two dimensions should be combined in a comprehensive microsimulation approach for a large coverage of FS&N issue. For nutrition, it is relevant to characterize households according to its members nutritional statuses (e.g., stunt children under 5 years old), and for food security, according to their food access (food purchasing power but also household diet diversity), food sufficiency (daily calories consumed compared to the average individual requirement), and food adequacy (nutrients intakes per capita per day compared to suggested requirements) dimensions. Household surveys and food composition tables are the basis to build FS&N indicators at the household level for this methodological approach.

The aim of this paper is to provide a comprehensive microsimulation approach tackling these challenges and allowing the ex-ante evaluation of policies on FS&N. The secondary objective is to apply the proposed method to the analysis of market incentive policies in Kenya’s FS&N.

The nutrition situation in Kenya has been improving in the last decade, but the progress is uneven, and the arid and semi-arid land (ASAL) regions still show very high stunting (above 30%) and wasting (15%) rates (Kenya National Bureau of Statistics, 2015). The current dietary intakes in Kenya show inadequate consumption of some food groups (i.e., vegetables, fruits, nuts and seeds) and nutrients (i.e., iron and folic acid) (Development Initiatives, 2018). The food security situation is also critical in the ASAL regions where 2.6 million people were estimated to be acutely food insecure in 2018 (Food Security Information Network, 2019). The current food insecurity is attributed to the frequent droughts, but also to the high costs of domestic food production, in part triggered by the high costs of inputs. The high global food prices and low purchasing power of a large proportion of the population living in poverty are also critical drivers (Kenya Agricultural Research Institute, 2012). Thus, the FS&N literature of Kenya highlights two dimensions of the problem, i.e., the quality of food consumption and the quantity of food access.

Against this situation, the Kenya Ministry of Devolution and Planning designed a working agenda to ensure progress in the attainment of SDG#2, planning programs to enhance the quality and quantity of food production, access and availability through increased agricultural productivity. Among the programmed interventions there are market access incentives such as local production of fertilizers or infrastructure investments to reduce transaction costs (Kenya Ministry of Devolution & Planning, 2017). This paper performs a microsimulation analysis of a market access improvement scenario (higher public investment on infrastructure, i.e., roads) in Kenya on food access, sufficiency, and adequacy at the household level, as a FS&N case study.

The paper is organized as follows. Section 2 presents the theoretical background of the analysis, Sect. 3 develops the methodological approach, and Sect. 4 presents the case study including data requirements for the application to the Kenya case study. Section 5 presents FS&N results for both benchmark and market access scenario. Section 6 discusses those findings in terms of their usefulness and need for agricultural (and related) policy recommendations to tackle the SDG#2. Section 7 concludes.

## Theoretical background

From modelling to data requirements, this section presents the relevant literature grounding the methodology and analysis of policies to tackle FS&N issues.

### The nature of the FS&N problems

Food insecurity and malnutrition are characterized by the multi-dimensionality (economic and non-economic) of its causes. Even when malnutrition is identified at the individual level, an inadequate diet (in calories and nutrients) and related diseases encompass structural problems, such as poverty, low educational coverage, inappropriate caring practices, unhealthy environment, food insecurity and unemployment, found at the household level but also at the population one (Keats et al., 2018). For those reasons, the case-by-case study (Akombi et al., 2017; Chiputwa & Qaim, 2016; Ecker et al., 2010), considering differences across households’ socio-economic and nutritional characteristics, their environment (region, sanitation, etc.) and pathways to policy impacts are the keys to tackle this multi-dimensional issue with a proper policy reform.

Even when nutritional information could help households’ heads to become aware of nutrients deficits (Byrd et al., 2017), the economic dimension of the food insecurity and malnutrition problem prevails. The resource constraints, the high costs of domestic food production, the volatility and the change in relative prices of food, determine both, the daily average caloric intakes and the dietary diversity of the households (Kenya Agricultural Research Institute, 2012). Deficiencies in micronutrients (e.g., vitamin A, iron, and zinc in East African countries) and macronutrients intakes (e.g., protein from animal sources in the SSA countries) are highly and positively correlated with calorie deficiency (Ecker et al., 2010). Furthermore, the demand of food rich in those nutrients display high income elasticities (Desiere et al., 2018).

### Modelling for FS&N-sensitive policy evaluation

Agriculture can impact FS&N outcomes through several pathways, that is, agriculture as a source of food, as a source of income for food and non-food expenditures, related agricultural policy and international food prices affecting food consumption (quantity and diet composition); and the role of women in farms and in household diet decisions (Kadiyala et al., 2014). Since the 90’s, benefiting from the seminal works of Deaton (1989a, 1989b, 1997), a vast empirical economic literature emerged on the change in agriculture income and prices on poverty impact by applying microeconomic approaches using household surveys. However, this literature only addresses purchasing power dimension without getting into the nutritional dimension of the FS&N problem. On the other side, a rich medical literature employs microsimulation approaches with individual and household surveys to develop FS&N indicators (e.g., Food Security Score, Body mass index, nutrients intakes such as potassium, sodium or fat) and considering pre-existent diseases (e.g., diabetes, cardiovascular diseases in adults, and caries epidemic in children) to evaluate health cost-saving of food policies (e.g., banned/taxes on sugar-based food and beverages; subsidies on fruits and vegetables). Nutrition and consumption quality is the core of this literature, but it does not account for any purchasing power dimension (Basu et al., 2013, 2018; Chi et al., 2014; Mozaffarian et al., 2018). Finally, the literature on macro-microsimulation models appears as a better combination for both, agricultural policy simulation and FS&N household impact (Cockburn et al., 2014). Simulation models (e.g., partial and general equilibrium models) evaluate the consequences of a policy shock over macroeconomic, average agents and sector variables, while linked microsimulation models allow to exploit households’ heterogeneity (i.e., preferences, endowments, members composition, anthropometric measures of its members, etc.) to also compute distributional effects on FS&N indicators. Nonetheless, economic papers based on this combined approach are still oriented to welfare and food access dimension (Beyene et al., 2018; Boulanger et al., 2020; Pauw et al., 2018), lacking the biological-nutritional dimension (i.e., macro and micro-nutrients; nutritional and health statuses of household members). Nechifor et al. (2021) is the recent exception quantifying the COVID-19 restrictions and policies impact on welfare, daily calories and macronutrients intakes per capita.

An appropriate approach to evaluate agricultural policy on food access (food purchasing power), food sufficiency (daily calories intakes and requirements) and food adequacy (diet diversity and nutrients intakes and requirements), needs a combination of policy simulation models and a microeconomic approach. This combination can only be achieved joining, knowledge of the biology-nutrition and economics disciplines to find and refine FS&N metrics at individual and household levels. These are the challenges to quantify the impact of agricultural policies with more completeness to the FS&N analysis (Qureshi et al., 2015). Even when assessments of agricultural interventions on nutrition outcomes highlight a scarce evidence to advise on the prioritisation among competing nutrition-sensitive agricultural policies (Masset et al., 2012), literature results point out that improving commercialisation channels (e.g., transport infrastructureAtack et al., 2009; Donaldson & Hornbeck, 2016; Zeller et al., 1998) could lead to positive impact on farmers’ income and food access (Chege et al., 2015), on nutrition (Carletto et al., 2017) and on diet diversity (Koppmair et al., 2017), enhancing access to higher-value nutritious foods, such as fruits, vegetables, and animal-source products, which are more perishable than staple foods (Muthini et al., 2020). Improving food access with region-specific public policies could tackle nutritional deficits locally avoiding intensifying elsewhere (Desiere et al., 2018; Ecker et al., 2010).

### FS&N indicators and data requirements

There is no single indicator to measure all FS&N dimensions. Therefore, when selecting FS&N indicators, a large coverage of the different aspects of the problem is required, and the standardization of metrics is strongly recommended for comparison.

Nutrition indicators are measured at the individual level and anthropometric measurements of children below 5 years of age are the most widely used, with standardized methods and greatest consensus on its interpretation (UNICEF & WHO, 2010; WHO, 2010). Among them, the most popular indicators are metrics of child wasting and child stunting, reflecting various dimensions of nutritional problems. Wasting (low weight for height) is a proxy of acute malnutrition particularly relevant to monitor acute food shortages and relief operations, while stunting (low height for age) is an indicator of chronic undernutrition result of long-standing adverse conditions, and thus, more appropriate to evaluate long-term effects of development interventions (De Haen et al., 2011).

Food security metrics can be collected at distinct levels: at both national and regional levels with the FAO food balance sheets (FBS) measuring undernourishment, and at the household level (and occasionally at the individual level) with food consumption modules of specialized household surveys. The latter allows to construct an array of FS&N indicators going beyond the calorie focus and to better understand the role of dietary quality (by independently measuring macro and micronutrients intake) and diet diversity, as well as the impact of policies in these important dimensions the FS&N problem (De Haen et al., 2011).

Food security can be measured at household level by direct outcomes of food consumption in terms of quantity and related calories per person per day (food sufficiency) or quality measured by nutrients intakes per capita per day (food adequacy), and also by indirect outcomes related to food access, availability and stability such as the food expenditure and diet diversity indicators (IPC Global Partners, 2012). These are FS&N dimensions that would largely cover the impact of agricultural policies, where the consideration of some qualitative aspects of the diet could be the pathway for nutrition improvement (Herforth & Ballard, 2016).

Thus, for the simulation of FS&N impacts of agricultural policies, the household consumption surveys properly combined with food composition tables are considered the best starting point for this analysis, and other useful econometric estimates, such the calorie-price and the calorie-income elasticities at national and regional levels, could also refine the path-through (De Haen et al., 2011; Ecker & Qaim, 2011; Rudolf, 2019; Santeramo & Shabnam, 2015; Yu & Shimokawa, 2016).

## Methodological approach

**Fig. 1:**
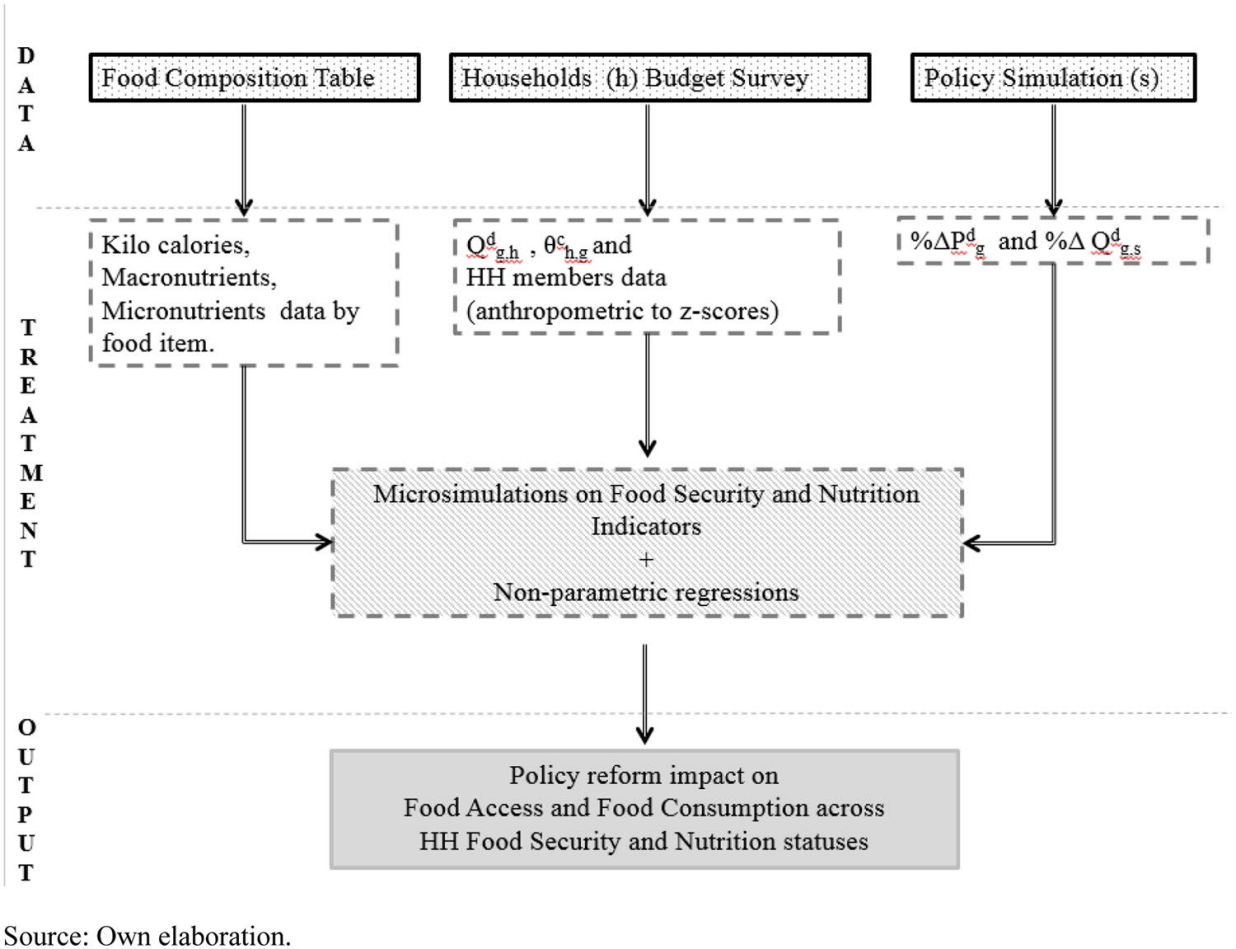
FS&N microsimulation methodology scheme. Source: Own elaboration

This paper provides a comprehensive FS&N microsimulation model for an ex-ante evaluation of policies targeting FS&N issues at the household level. This model is combined in a top-down fashion. A policy simulation model simulates a policy scenarios at the real level of their implementation (national or sectoral). Some steps are essential in the definition and implementation of the methodological approach. First, the definition of the relevant FS&N indicators and the data collection to construct them at the household level. Second, the evaluation of the availability of data sources and their treatments for consistency. Finally, the linkages between the FS&N microsimulation approach based on these indicators and the requirements for its implementation for a policy evaluation. Figure 1 shows the scheme of this methodological approach.

This approach requires three inputs. Firstly, the impacts on food price and food consumed quantities due to a policy shock and two datasets (Fig. 1). The first insight needs an external model, either an econometric or a general or partial equilibrium simulation approach, that could provide the changes in food prices and food consumed quantities due to the implementation of a given policy reform. These results are then introduced, as exogenous changes, in the microsimulation module. For the application case included in this paper, a Computable General Equilibrium (CGE) model provides this first input. The CGE model is a comparative static variant of the STatic Applied General Equilibrium model (STAGE) (McDonald, 2021) and its extension for the context of developing countries (STAGE-DEV) (McDonald et al., 2016). In particular, STAGE-DEV accounts for the non-separability of the dual roles of smallholders as producers and consumers. Subsistence farmers produce their Home Production for Home Consumption (HPHC) allocating labour and capital for own consumption. Further details of this CGE model are provided in next section an in the Online Annex A.

Secondly, a household budget survey providing data on food consumption by food item (quantities and expenditure) at household level. Ideally, the survey should provide anthropometric measures at the household’s member level and other households’ characteristics (e.g., size, location, income) useful for data/results analysis. Thirdly, a food composition table containing the biochemical composition, i.e., kilocalories and nutrients per 100 g of edible quantity of each food item. Their combination allows to compute the FS&N indicators for a given point in time (the FS&N benchmark situation) and the shares required in Eqs. (1) to (3) to perform microsimulations. The three inputs must be consistent in terms of food item coverage and spatial disaggregation.

Finally, since FS empirical literature (Abdulai & Aubert, 2004; Salois et al., 2012) supports a non-linear and non-monotonic relationship between energy/nutrients per capita intakes and households’ characteristics such as income per capita, we run non-parametric smoothing regressions^1^ of previous food access and food consumption (sufficiency and adequacy) microsimulation results across economic and FS&N households’ characteristics.

### Food security and nutrition indicators

The FS&N indicators characterize households in terms of food access, food consumption (sufficiency and adequacy) and nutrition status, which represent the benchmark for microsimulations of a policy scenario. Table 1 summarizes the FS&N indicators used in the paper.

**Table 1:** FS&N indicators at the household level

Dimension	Outcomes	Measured by	Indicators used in this study:
Food security	Direct outcomes—Food consumption		
	*In terms of calories* *(sufficiency)*	Total calories consumed	Dietary Energy Consumption (**DEC**) per capita per day
	*In terms of macronutrients* *(adequacy)*	Caloric contribution of the different macronutrients (in Kcal and % of DEC)	Caloric contribution of **proteins** to total calories per capita per day
			Caloric contribution of **fats** to total calories per capita per day
			Caloric contribution of **carbohydrates** to total calories per capita per day
	Indirect outcomes—Food access		
	*In monetary terms*	Food expenditure	Total food expenditure in the household and per capita per day
	*In qualitative terms*	Household dietary diversity	Household Consumption and Expenditure Surveys—Dietary Diversity Score (**HCES-DDS**)
Nutrition	*Stunting (HAZ* < *-2)*	Height for age z score (HAZ) in children below 5 years of age	Stunting defined as **HAZ** < -2
			Minimum HAZ registered in the household
			Proportion of stunted children below 5 years of age suffering in the house

Source: Own elaboration

#### Food sufficiency (calories)

To evaluate the quantity of food consumed, we calculate the calories consumed using the Dietary Energy Consumption (DEC) per day per capita.

#### Food adequacy (macronutrients)

To assess the diet in terms of macronutrients, we calculate the calories obtained from each of the energy-yielding macronutrients (fat, proteins and carbohydrates) per day and per capita following the methodology described by the Food and Agriculture Organization of the United Nations (FAO)to be used with the ADePT-FSM software (Moltedo et al., 2014, 2018).

#### Food expenditure

The food expenditure is computed per day at the household level and per capita according to the household composition. This indicator is a measure of the purchasing power of food and thus a good indicator of food access.

#### Household dietary diversity score

Beyond the sole calories consumed, literature highlights the importance of analysing the quality of consumption in developing countries (Donini et al., 2016; Ruel et al., 2013; Kennedy et al., 2007, 2010; Hoddinott & Yohannes, 2002). As the 2015/16 Kenya Integrated Household Budget survey (KIHBS) (Kenya National Bureau of Statistics, 2018) collects food consumption data for a 7-days period, we use the household consumption and expenditure surveys dietary diversity score (HCES-DDS), an indicator proposed by the FAO, which can be used for longer reference periods (Moltedo et al., 2018).

#### Stunting

The z scores are calculated comparing the height of the measured child with the mean of the height of children with same sex and age from a reference population. When the z-score for height for age (HAZ) is below − 2 the child is identified as being stunted (suffering stunting), and below − 3 severely stunted. The calculation of these indicators employs the Anthro Software, using the WHO Child Growth Standards as the reference population (WHO, 2019). From this indicator, the lowest HAZ is defined as the household minimum HAZ. It stands for a proxy of the presence of stunting in the household. Besides, we calculate the proportion of stunted children under five out of all children under five living in the household.

### Food security and nutrition microsimulation approach

The FS&N microsimulation evaluates the impact, at the household level, of wide (national, regional) economic policies to improve food access and food consumption (sufficiency and adequacy). Any implemented policy reform causes changes in food prices and households’ food consumed quantities, modifying the initial households’ food access and food consumption patterns– in quantity and quality.

The policy impact evaluation of FS&N dimensions measures, first, the change in the accessibility of food in the base of its affordability: food access. Thus, Eq. (1) shows, given the expenditure shares (${\theta }_{i,h}$) of each food item ($i$) in each household ($h$), the food purchasing power ($fe$) changes when prices of food items (${p}_{i}$) vary due to a policy implementation.

1 $$\Delta fe_{h} = \mathop \sum \limits_{i = 1}^{n} \left( {\theta_{i,h} \cdot \Delta p_{i} } \right)$$

Secondly, the consumption of edible food (in grams) in a household also changes because of a policy reform. Thus, the daily DEC per capita (${dec}_{h}$) and the macronutrients ($j$) intakes per capita per day (${mac}_{j,h}$) is affected by changes of food price and availability. Given the energy shares (${\varnothing }_{i,h}$) of each food item in the daily diet per capita in a household, equation (2) computes the change in DEC (kcal. per capita per day) at the household level when consumed quantities (${q}_{i,h}$) of each food item change in a household.

2 $$\Delta dec_{h} = \mathop \sum \limits_{i = 1}^{n} \left( {\emptyset_{i,h} \cdot \Delta q_{i,h} } \right)$$

Knowing the fat, protein, and carbohydrate composition of each food item (kcal. per 100 g), we compute the contribution of each macronutrient $j$ in the energy provided by each food item (${\gamma }_{j,i}$). Equation (3) measures the change in each macronutrient intake per capita per day when consumed quantities of each food item does in the household.

3 $$\Delta mac_{j,h} = \mathop \sum \limits_{i = 1}^{n} \left( {\gamma_{j,i} \cdot \Delta q_{i,h} } \right)$$

Equations (2) and (3) measure household food consumption, sufficiency and adequacy, respectively, as a result of a given policy reform affecting food price and production.

## Case study: Kenya

The specific FS&N microsimulation presented in this paper evaluates the impact of an improvement in market access conditions in Kenya using three original insights: (i) the 2015/2016 KIHBS that collects economic and FS&N variables (anthropometric measurements), (ii) the biochemical composition, energy, and nutrient yield of food consumed items taken from the Kenya Food Composition Tables (FCT KEN2018) and (iii) the change in food prices and food consumed quantities from a policy simulation model developed for Kenya.

For the last insight, the STAGE-DEV CGE model (McDonald et al., 2016) simulates an improvement in market access due to public investment in infrastructure on domestically traded commodities (Boulanger et al., 2018, 2020). Investments to improve infrastructure leads to lower transaction costs associated with agricultural activities with a potential to reduce the cost of accessing inputs (Dercon et al., 2009). Better infrastructure and improved market access can thus reduce consumer prices, increase the share of value added accruing to farmers and increase domestic production of marketed commodities. This scenario is simulated within a CGE framework consistent in terms of data sources and sectoral/geographical details for Kenya. The model is calibrated with a Social Accounting Matrix (SAM) of Kenya in 2014 (Mainar-Causapé et al., 2018, 2020). Under this scenario, the domestic trade and transport margins fall by 30% in exchange for an investment of 4 billion of Kenyan Shillings to improve infrastructure, financed by government savings. The cost to transport fertilizers input decrease by 30%; as better roads would make their delivery cheaper (Minten et al., 2013). Farmers will then increase the use of fertilizers and increase their productivity. In addition, trade costs of agricultural commodities would fall by the same amount. Even in this case, the magnitude of the shock looks plausible (Key et al., 2000), in particular given the amount of the investment planned and the size of trade margins in the country (Boulanger et al., 2020). Moreover, the elasticities estimated by Benin et al. (2008) support the size of this shock.^2^

The implementation of the FS&N microsimulation approach to evaluate the impact of the market access improvement requires building the FS&N benchmark for Kenyan households. The following household survey and the food composition information have been treated to become compatible in terms of food items.

1. The 2015/2016 KIHBS provides the consumption quantities by food item, source of origin, total and food expenditure among other characteristics at the household and member levels (Kenya National Bureau of Statistics, 2018). This survey provides information for 21,773 households.
2. The 2018 Kenya Food Composition Tables (FCT KEN2018) provides biochemical contents (energy and nutrients) of food items allowing for the calculation of FS&N indicators when combined with the quantities consumed in each household (FAO & Government of Kenya, 2018). To match the nutrient contents in the FCT to consumed food items collected in the 2015/2016 KIHBS, we construct the Kenya Nutrient Content Table (KNCT) following Moltedo et al. (2014, 2018).

Once set up the FS&N benchmark, the non-parametric regressions of microsimulation results are performed across economic (the per capita expenditure -in log- distribution), food security (the per capita DEC -in log- distribution; HCE-DDS), and nutrition (the distribution of height for age in the household, HAZ) statuses of households in the benchmark situation. Moreover, all results are presented at the national and geographical areas defined as Metropolis (Nairobi and Mombasa), other urban (but Metropolis) and rural areas.

The sample includes 21,625 households at the national level, which are geographically distributed in 1003 households in the Metropolis areas, 7586 households in other urban areas and 13,036 households in the rural areas. The national average is around four persons per household without distinctions of households’ composition in terms of age and/or gender. Comparatively across the geographical distribution, metropolitan households have fewer household members than the rest of the country (Table 2).

**Table 2:** Descriptive statistics of the population of Food Access and Food Consumption indicators (national and geographical areas)

	National			Metropolis			Other Urban			Rural		
	N = 21,625			5%			35%			60%		
	mean	p50	sd	Mean	p50	sd	mean	p50	sd	mean	p50	sd
HH size	4.28	4	2.52	2.98	3	1.9	3.79	3	2.44	4.67	4	2.53
Food access indicators												
Food Expenditure (share)	0.56	0.56	0.19	0.4	0.4	0.14	0.49	0.49	0.18	0.64	0.65	0.17
Food Expenditure (per day per capita)	125	97	186	193	161	132	145	115	202	108	84	178
HCE-DDS	11	11	3	12	12	3	11	11	3	10	11	3
Food Consumption Indicators												
DEC (kcal. per capita per day)	1970	1769	1054	2058	1906	1038	1983	1801	1030	1938	1702	1068
Fat (kcal. per capita per day)	475	403	316	565	502	345	505	437	324	435	361	295
Share	*0.24*	*0.23*	*0.08*	*0.27*	*0.26*	*0.08*	*0.25*	*0.24*	*0.09*	*0.22*	*0.21*	*0.07*
Protein (kcal. per capita per day)	220	191	132	246	218	141	223	200	130	211	180	129
Share	*0.11*	*0.11*	*0.03*	*0.12*	*0.12*	*0.03*	*0.11*	*0.11*	*0.03*	*0.11*	*0.11*	*0.02*
Carbohydrate (kcal. per capita per day)	1266	1138	679	1232	1147	614	1242	1125	654	1287	1141	708
Share	*0.65*	*0.66*	*0.09*	*0.61*	*0.61*	*0.09*	*0.63*	*0.64*	*0.1*	*0.67*	*0.68*	*0.08*

*Source* Own elaboration from 2015/2016 KIHBS and 2018 KNCT

Food access indicators show the food expenditure at the household level and the household diet diversity score based on that food expenditure. At the national level, households spend on average around 60% of their income on food, consuming 11 out of 16 household diet diversity score food groups on average. However, this pattern differs across areas: rural households spend 64% of the budget on food while metropolis households only 40%. In terms of the (deflated) daily amount spent on food per capita, Mombasa and Nairobi almost double the amount spent in rural areas, and diet diversity according to food expenditure is also greater in the metropolis (Table 2).

Besides, at national level, households spend around 21% of food expenditure on bread and cereal, 10% on meat and 10% on milk, cheese and eggs. Thus, a decrease in prices of these food items might cause a considerable improvement in their purchasing power.

The daily DEC per capita and their related macronutrients intakes (fat, protein, and carbohydrate) shows the food consumption patterns in Kenyan households (Table 3). The national average of daily energy consumed per capita is 1970 kcal with a right-skewed distribution. Average daily DEC per capita is similar across areas; however, the rural areas display more dispersion in energy consumption per capita per day.^3^ With reference to the average macronutrients intakes per capita at national level, the shares of caloric intakes provided by fats, proteins and carbohydrates are 24%, 11% and 65% respectively. The metropolitan areas display the highest caloric intake provided by fats (27%) while the rural areas show the highest shares of carbohydrates (67%). The standard deviations do not show significant differences among regions, although in the case of the DEC per capita the standard deviation is relatively high (1054 at national level, 1038 urban and 1068 rural).

**Table 3:** Proportion of the households within, below or above the ranges of population macronutrient intake goals^a^ by areas in Kenya

	National (%)	Metropolis (%)	Other urban (%)	Rural (%)	Min HAZ < = − 2 (%)
A balanced diet	41.42	40.78	40.83	41.81	39.14
A diet that does not meet any of the three recommended	5.01	9.37	6.04	4.08	2.96
goals for energy-supplying macronutrients					
Dietary energy provided by protein below	39.63	29.01	37.36	41.78	46.82
the lower recommended threshold (10%)					
Dietary energy provided by fat below	12.38	7.58	10.40	13.90	14.16
the lower recommended threshold (15%)					
Dietary energy provided by carbohydrate below	10.56	22.43	13.91	7.69	5.62
the lower recommended threshold (55%)					
Dietary energy provided by protein above	5.28	10.87	6.38	4.21	3.18
the upper recommended threshold (15%)					
Dietary energy provided by fat above	17.71	29.11	21.38	14.69	13.30
the upper recommended threshold (15%)					
Dietary energy provided by carbohydrate above	10.92	6.18	8.59	12.64	14.46
the upper recommended threshold (55%)					

The ranges of population nutrient intake goals for energy-supplying macronutrients are expressed as percentage of energy: fat (15–30%), carbohydrate (55–75%) and protein (10–15%)

*Note* Column Min HAZ < = − 2 refers to the proportion of households with at least one stunted child under 5 years old

*Source* Own elaboration from 2015/2016 KIHBS and 2018 KNCT

These shares of macronutrients intakes fall within the ranges that the FAO and the WHO suggest for a balanced diet to avoid related diseases (WHO, 2003). At national level 59% of households has unbalanced intakes of macronutrients (Table 3). Around 3% of households (national level) does not meet the recommendations for any of the macronutrients, and 12%, 39.6% and 10% of households at the national level do not meet the minimum percentage recommended by FAO for fats, proteins and carbohydrates respectively. In relation to diet energy provided by macronutrients above the upper recommended threshold, results of the survey underline a high percentage of households consuming energy from fats, i.e., 29% in Metropolis, 21% in Other Urban and 15% in the rural areas.

The analysis of the FS&N benchmark in Kenya also reveals that, although poor households spend more of their income on food than rich ones, on average the household diet diversity (HCE DDS) increases with the livelihood in all the areas of the country. Moreover, the energy availability at the household level (DEC) is positively associated with the diet diversity score according to the food expenditure (HDDS-HCE). In terms of macronutrients, the proportion of carbohydrates decreases with the increase of diet diversity, while fats and proteins proportion increase.^4^

For nutrition analysis, the 2015/2016 KIHBS (Kenya National Bureau of Statistics, 2018) includes a sub-sample of 7530 households with anthropometric measures which allows computing the percentage of children with low height for age for selected households, the minimum HAZ and the proportion of stunted children per household. According to this sub-sample, 31% of the households present at least one stunted child (min HAZ lower than − 2). Moreover, 10% of the households display up to half stunted children and 19% more than half children with growth retardation or stunting. Within households with at least one stunted child, macronutrients deficits are even worse compared to the national average, i.e., 46.8% of these households fall below thresholds of calories provided by proteins, and 14.2% are under fat intakes recommendation (Table 3).

## FS&N results of improving market access in Kenya

As suggested by the economic literature (Boulanger et al., 2018, 2020; Dercon et al., 2009; Key et al., 2000; Minten et al., 2013), an investment to improve of market access via new and improved roads reduces consumer prices of agricultural commodities and fosters their consumption (Table 4). Nonetheless, the shock does not impact equally across products and geographical areas due to the heterogeneity of market conditions and households’ preferences recorded by the Social Accounting Matrix (SAM), the database on which the CGE is calibrated, and the behavioural configuration of the model. On average, lower transaction costs lead to greater price reduction on vegetables, fruits, roots and tubers, and bread and cereals, which are the commodities with higher margins and, consequently, whose production increases the most. Changes in consumption depend on two main factors. First, the initial level of consumption recorded in the SAM, derived from the 2015/16 Household Budget Survey. Second the estimated elasticities of each household's income (Vigani et., 2019). The increase in consumed quantities results on average greater in rural than in Metropolis areas. In contrast, for meat, fish, and seafood lower prices lead on average to a greater consumption in the Metropolis compared to the rest of the country. These results display differences in the consumption impact and potential changes in consumption patterns across areas. These differences are also reflected in the food access and food consumption (calories and nutrients) consequences due to this policy at the household level.

**Table 4:** Market Access Improvement (average % changes for prices and consumed quantities)

	Average % changes				
	Prices	Consumed quantities			
		National	Metropolis	Other Urban	Rural
Beer	− 0.278	0.182	0.232	0.245	0.142
Bread and Cereals	− 0.994	1.184	0.549	1.287	1.173
Coffee, tea and cocoa	− 0.278	0.185	0.248	0.250	0.141
Fish and seafood	− 0.024	− 0.315	0.002	− 0.407	− 0.286
Food products n.e.c. Spices & Miscellaneous	0.066	− 0.480	− 0.052	− 0.496	− 0.516
Fruits	− 0.790	1.293	0.676	1.409	1.303
Meat	− 0.134	− 0.098	0.083	− 0.090	− 0.131
Milk, cheese and eggs	− 0.332	0.301	0.290	0.376	0.253
Mineral water, soft drinks, fruit and vegetable juices	− 0.278	0.217	0.231	0.251	0.174
Oils and fats	− 0.862	1.258	0.639	1.421	1.216
Roots and tubers	− 0.879	1.592	0.787	1.694	1.600
Spirits	− 0.278	0.168	0.257	0.243	0.120
Sugar, jam, honey, chocolate	− 0.221	0.094	0.190	0.112	0.075
Vegetables	− 1.474	2.860	1.239	3.120	2.873
Wine	− 0.278	0.223	0.250	0.252	0.149

*Source* own CGE model’s results

As suggested by the economic literature (Boulanger et al., 2018, 2020; Dercon et.al, 2009; Key et al., 2000; Minten et al., 2013), an investment to improve of market access via new and improved roads reduces consumer prices of agricultural commodities and fosters their consumption (Table 4). Nonetheless, the shock does not impact equally across products and geographical areas due to the heterogeneity of market conditions and households’ preferences recorded by the Social Accounting Matrix (SAM), the database on which the CGE is calibrated, and the behavioural configuration of the model. On average, lower transaction costs lead to greater price reduction on vegetables, fruits, roots and tubers, and bread and cereals, which are the commodities with higher margins and, consequently, whose production increases the most. Changes in consumption depend on two main factors. First, the initial level of consumption recorded in the SAM, derived from the 2015/16 Household Budget Survey. Second the estimated elasticities of each household's income (Vigani et al., 2019). The increase in consumed quantities results on average greater in rural than in Metropolis areas. In contrast, for meat, fish, and seafood lower prices lead on average to a greater consumption in the Metropolis compared to the rest of the country. These results display differences in the consumption impact and potential changes in consumption patterns across areas. These differences are also reflected in the food access and food consumption (calories and nutrients) consequences due to this policy at the household level.

### Food access

Non-parametric regressions have been estimated between the change in food security indicators (microsimulations) and households’ characteristics, such as expenditure per capita, DEC per capita, HCE-DDS, and nutritional status for children under 5 years old—stunting.

The change in households’ food access and affordability is computed as the traditional food consumption effect. Given the composition of each household’s basket of food (food expenditure shares), it measures the consequences in the food purchasing power for each household of the change in food prices due to the improved market access.

**Fig. 2:**
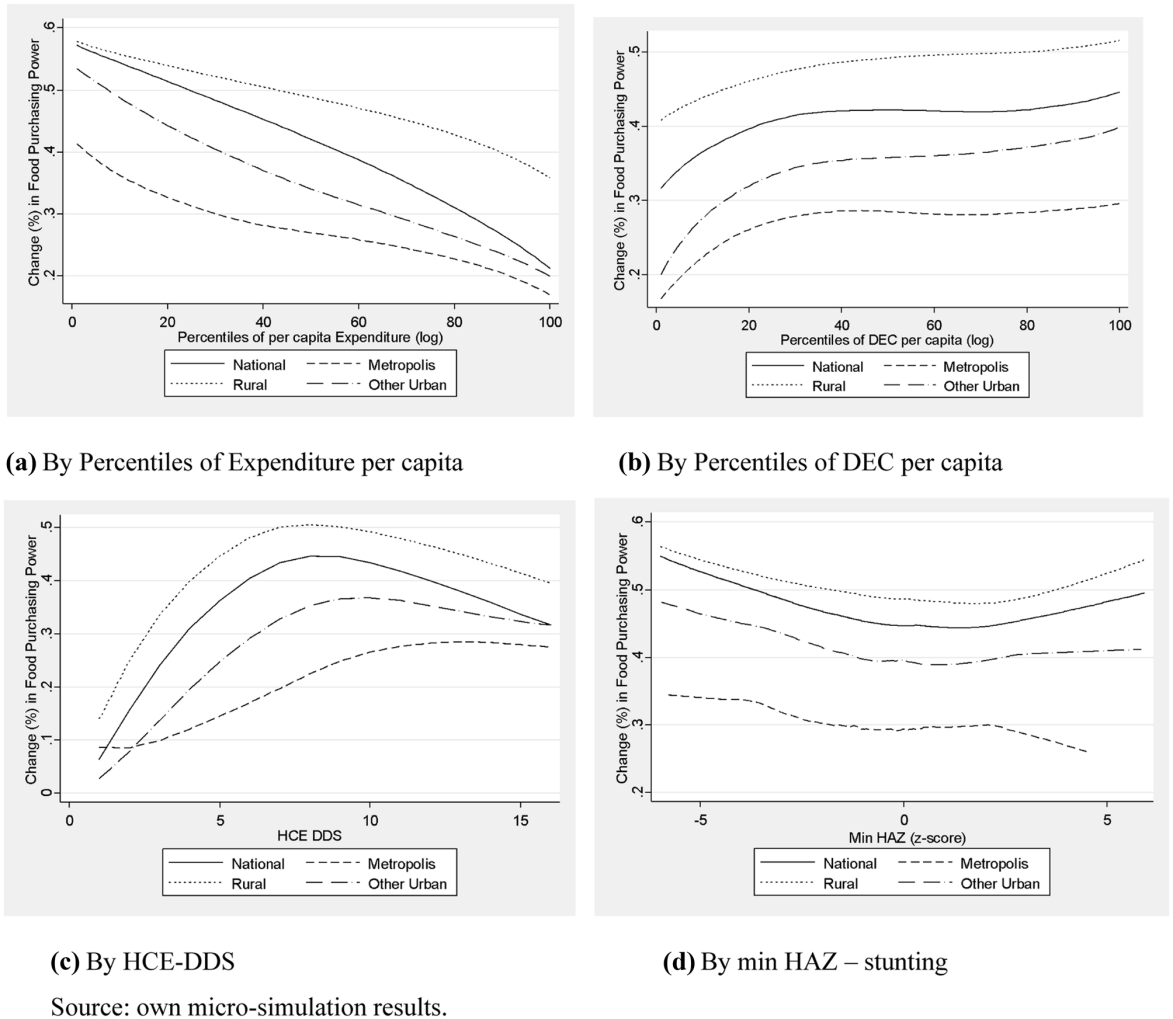
Food access impact of improving market access (% change in purchasing power of food)

Figure 2 presents the households’ change in purchasing power of food, at the national and geographical level, across percentiles of per capita expenditure (panel (a)), percentiles of DEC per capita (panel (b)), dietary diversity (HCE DDS, panel (c)) and the lowest z score for height for age in the household (height for age z score, HAZ, panel (d)).

The Market Access scenario generates a positive food access effect for all the Kenyan households. However, the improvement in food access is greater for the rural households compared to both Metropolis and the other urban areas. Differences in the benchmark food access situation and the households’ preferences explain these disparities across households/areas. This ranking across areas prevails under all food security characteristics and anthropometric measures of households.

Moreover, this positive change in food access declines with the livelihood leading to a pro-poor improvement of food consumption in all areas (Fig. 2a). This is a desirable food security outcome from a policy reform in terms of the food access dimension.

Additionally, this food consumption effect increases with the daily energy consumed per capita (Fig. 2b). In other words, food purchasing power increases with the benchmark distribution of the DEC per capita across households. Although this result seems in contradiction with the previous one (panel (a)), one should consider that poor households do not necessarily display an appropriate diet, sometimes extremely high in calories and not correctly balanced in terms of nutrients.

Considering the benchmark of the diet diversity of households, the food purchasing power increases with the increment of diet diversity measured by the HCE DDS (Fig. 2c). However, this relation displays an inverted U-shape form achieving a maximum positive impact for households with an average diet diversity (HCE DDS = 8 at the national level). Looking at the distribution boundaries, the households with the highest score of diet diversity display a greater improvement in food purchasing power compared to those with the lowest diet diversity score.

Finally, according to the anthropometric indicators of children under 5 years old (Fig. 2d), the households with at least one stunted child (HAS < − 2) benefit from a greater food purchasing power increase compared to the households without malnourished children. The food access effect remains greater in the rural areas but the gap effect between households with and without at least one stunted child is greater in the urban areas, both Metropolis and Other urban areas.

Overall, these outcomes are desired when improving food market access conditions. The impact evaluation of this policy scenario at the household level allows identifying and isolating the consequences on the food access dimension for the most vulnerable cases in terms of food insecurity (e.g., rural households, poor households all over the country, and households with chronic malnourished children).

### Food consumption

**Fig. 3:**
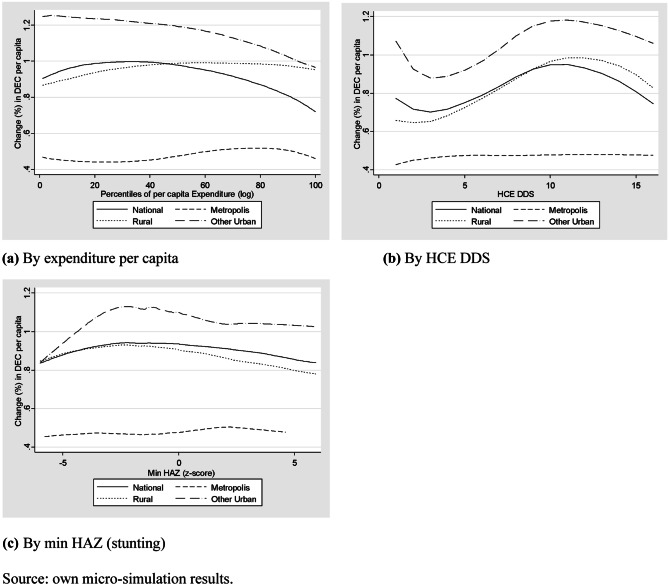
Food sufficiency impact of improving market access (% change in DEC per capita). **a** By expenditure per capita, **b** By HCE DDS, **c** By min HAZ (stunting). Source: own micro-simulation results

Food consumption impact at the household level is measured in terms of food sufficiency, considering the change in the DEC per capita, and food adequacy, computing the change in macronutrients intakes per capita. Figure 3 presents the results of non-parametric regressions of the change in DEC per capita across the expenditure per capita, the household diet diversity score and the min HAZ at the household level.

As for food access, the change in the DEC per capita is positive for all households. In contrast to the food access and affordability dimensions, the changes in the DEC per capita are not systematically greater for the rural households. Those households that increase the most their daily caloric intakes live in the urban areas different from the Metropolis.

Across percentiles of per capita expenditure, the poorest households at the national level display a greater increase in the DEC per capita compared to the richest ones. However, the households in the middle of the income distribution display the maximum positive impact for DEC per capita increase when improving market access conditions. This result differs across areas; for instance, whereas the change in the DEC per capita increases with the livelihood in rural areas, it declines in other urban areas (Fig. 3a).

The households with medium–high diet diversity (HCE-DDS = 11 at the national level and in other urban areas, and HCE-DDS = 13 in rural areas) increase the DEC per capita the most. In the Metropolis, the DEC per capita systematically rises with the increment of household diet diversity (Fig. 3b).

Summarising the food sufficiency outcomes from a market access scenario, the DEC per capita increases the most in poor households of other urban areas, with an average diet diversity and with at least one stunted child under 5 years old.

The increase in the DEC per capita does not necessarily imply an improvement in the quality of the diet. Therefore, it is important to analyse the change in macronutrients intakes per capita. Market access improvement allows increasing the consumption of all macronutrients (fats, carbohydrate, and protein) on average. However, this increase in macronutrient consumption differs across households according to their per capita expenditure, daily energy consumption per capita, diet diversity at the household level and children’s nutritional characteristics.

**Fig. 4:**
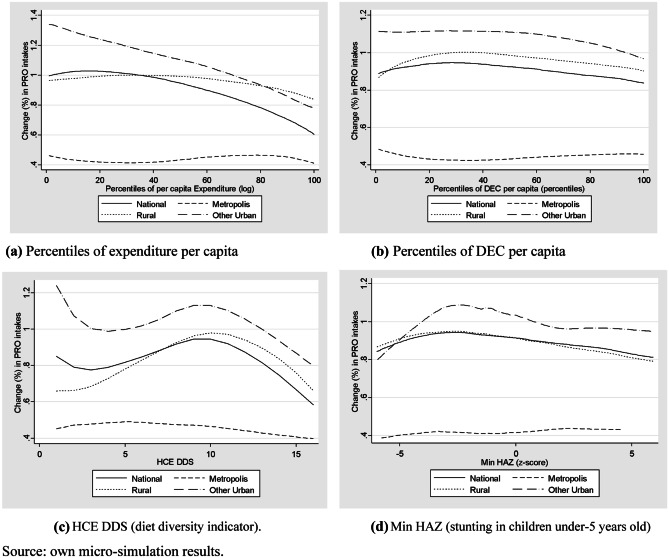
Protein effect (% change in Daily Proteins intakes per capita). (**a**) Percentiles of expenditure per capita, (**b**) Percentiles of DEC per capita, (**c**) HCE DDS (diet diversity indicator). (**d**)(Min HAZ (stunting in children under-5 years old). Source: own micro-simulation results

Even if results are available for all macronutrients, Fig. 4 presents only protein intakes, as unbalanced diets with low protein consumption are among the most frequent ones (39.63% of national households and 41.78% of rural households are below FAO’s recommendations) and within households with stunted children under 5 years old (46.82%) (Table 4).

As for the impact on DEC per capita, the change in protein intake per capita at the household level displays the greatest average impact in other urban areas, and the lowest in the Metropolis (Mombasa and Nairobi).

Across percentiles of per capita expenditure, the increase in proteins intakes per capita display a pro-poor impact in other urban areas, in line with results at national level. Nonetheless, in the rural and metropolitan areas the increase in protein intakes is slightly pro-rich (Fig. 4a).

The change in protein intakes per capita slightly increases with the DEC per capita in all areas (Fig. 4b). According to the household diet diversity in the benchmark based on the HCE-DDS indicator, the greatest increase in protein consumption per capita is for households with medium–high scores of diet diversity (HCE-DDS = 10 on average) in all areas (Fig. 4c).

Considering the minimum HAZ score for households with children under five years (Fig. 4d), the greatest increase in protein intakes per capita is in households that live in other urban and rural areas, and which have at least one stunted child under 5-year-old (HAZ < − 2). In the metropolitan area, the increase in protein intakes per capita is higher in households without stunting.

The impacts for fats and carbohydrates (Online Annex B) display comparable results as for proteins. Under this market access scenario and among the three macronutrients analysed, the average increase is greater for carbohydrates, followed by proteins. Other urban and rural households are those who benefit the most from this increase in macronutrients intakes and, also those who display more than half of their children with stunting.

## Discussion and policy implications

Providing policymakers with comprehensive ex-ante analyses of policy changes represents a critical task for applied economists. It requires the use of complementary tools assessing uneven and dynamic effects, confronted with the availability of timely and extended microdata.

### Methodological contribution

Addressing FS&N issues is challenging due to the multi-dimensional aspects of causes to be tackled. Policies are defined and implemented at national or regional levels, and economy-wide models are well suited to assess the effects of policy changes on the whole economy. At the same time, microsimulations are required to capture impacts on FS&N indicators at the pertinent individual or household levels. To analyse FS&N issues, linking top-down macro and micro approaches becomes fundamental to provide a comprehensive assessment.

This paper presents an original methodology that could be extended to analyse FS&N issues in any country context with the required availability of data. The approach is tested analysing the effects of an improvement of Kenyan infrastructure, which reduces transport costs and allows an improved market access. This policy is presently at stake in most developing countries. Kenya grants a perfect test case because of recently published food composition table for this country and the collected data for households that includes anthropometric measures for children and detailed consumption survey. These data are essential to build FS&N indicators at the households and individual levels covering economic and nutritional dimensions of this problem.

### Policy contribution

A CGE model provides the effects of the national policy on commodities price and quantities consumed. Investments in improving and building new roads reduce the prices of agri-food products benefitting domestic consumers. Rural areas benefit (increase consumption) of key food products (vegetables, fruits, roots and tubers, and bread and cereals) and metropolitan areas present greater consumption of meat and fish. Despite their usefulness, CGE results are not well suited to tackle FS&N issues at household level.

Selected FS&N indicators shed light on the differences across households in terms of their food access, food consumption and nutrition statuses. Results show uneven effects at macro and micro levels across the proposed household breakdown. FS&N microsimulation results suggest that better market access conditions improve food access effect for all households in Kenya, benefiting more rural households and most vulnerable households (i.e., poor and households with chronic malnourished children), showing a pro-poor improvement of food consumption in all areas. A greater market access is a pro-poor food and nutrition policy. In terms of food sufficiency, the DEC per capita of the poorest households increases more than the DEC of the richest ones. Market access improvement raises the consumption of all macronutrients (fats, carbohydrate, and protein) on average with significant differences across households according to their per capita expenditure, daily energy consumption per capita, diet diversity at the household level and children’s nutritional characteristics Furthermore, original data on households with children under five years highlight that those households require targeted policy support, mostly in metropolitan areas. Microsimulations help to identify which household benefits -or not- from a macro policy when national estimates (improvement) masks sub-national differences (relative deterioration).

Quality of data, heterogeneity within a sub-group or counterintuitive dynamics should be taken with caution. For instance, increase in the DEC per capita and quality of the diet can change in an opposite way. The increase in protein intakes appears slightly pro-rich in metropolitan areas. Generating detailed results on expected change by groups of households, on a geographically, socio-economic, or nutritional basis helps to define pertinent accompanying measures. These measures are crucial in addressing micro food and nutritional deficiencies. Indeed, the definition of a pertinent policy mix, e.g., including crop improvements and other relevant supply side policies, goes beyond the sole macroeconomic plan. The whole decision-making cycle also includes health, social or education measures.

Overall, most of the outcomes of the simulated policy are positive, confirming that strengthening market access is a promising strategy to tackle food insecurity. In addition, the proposed methodology allows identifying and isolating the consequences of policy change on the food access and consumption dimensions of the most vulnerable cases (e.g., rural households, poor households all over the country, and households with chronic malnourished children).

## Conclusions

Kenya, such as many other African countries, is concerned about the achievement of the Sustainable Development Goal #2 (SDG#2). Empirical evidence about food security and nutrition in Kenya accounts for deficiencies in food access, sufficiency, and an inadequate diet in terms of daily per capita calories and nutrients intakes. These nutritional deficiencies are among the causes of all forms of malnutrition in young population (e.g., stunting, wasting, and overweight) that can lead to impaired cognitive development, limited immunity to diseases, low educational performance, higher risk to chronic diseases, and even increased mortality of children.

FS&N problems are characterized by multi-dimensional causes and heterogeneity across households regarding their income and food expenditure, education of households’ head, regional sanitation coverage, the environment (access to potable water/wastewater system) and public policies with direct or indirect impact on the households’ nutritional status.

This paper provides a comprehensive method to assess impacts of public policies on FS&N indicators at the household level. The criteria provided by the literature and the data availability concerning food consumption and nutrition are key for choosing pertinent FS&N indicators. With an application to a market access improvement scenario on Kenya, this methodology focuses on the economic factors that affect Kenyan households’ food access and affordability, food sufficiency and macronutrient consumption, as a first consideration of food adequacy. This approach appears useful for the identification of agricultural/food policies that could help improving FS&N conditions in critical zones of the country (e.g., rural) or most vulnerable population groups (e.g., households with stunted children). The proposed methodology can be applied to any countries with comprehensive household surveys on consumption and nutritional aspects, coupled to food composition tables. This paper can further be seen as an application of this approach, proposing food item matching and consistency across datasets.

Results contribute to the discussion on how public policies can tackle the SDG#2. In terms of food access, policy effects are greater in rural areas, and households with at least one stunted child (HAS < − 2) benefit the most. This calls for targeted accompanying actions towards households without malnourished children, especially in urban areas. In contrast, changes in DEC per capita are not systematically greater for rural households. Those households that increase the most their daily caloric intakes live in urban areas. Food adequacy or macronutrients intakes per capita is improving unevenly, across households and areas. Overall, average increase is greater for carbohydrates and proteins. This paper provides a better understanding of the magnitude in changes by households, areas and macronutrients intakes. To sum-up, it shows that nutrition-sensitive agricultural policies, such as improving market access via infrastructure development, improve food security and nutritional indicators. Nonetheless, there is not a unique policy instrument but a set of tools that would be required to cover the diversity of households and territories suffering food consumption and nutrition deficiencies.

## Supplementary Information

Below is the link to the electronic supplementary material.

Supplementary file1 (DOCX 91 kb)

## Footnotes

1 Non-parametric regressions fit a local relationship between two variables, e.g., the change in DEC per capita (*y*- variable) and the log of per capita expenditure (*x*- variable). Non-parametric smoothing techniques are flexible tools for analysing unknown relationships between variables.

2 To show the robustness of the findings to changes in the value of the shocks, Annex C includes results where the reduction of trade and transportation margins are reduced by 15% and 45% respectively. The whole set of results is available upon request.

3 For the computation of the DEC we only consider food consumed at home. Tobacco and other stimulants were deleted as well as food consumed in restaurants and canteens.

4 The full set of data is available upon request to the authors.

## References

[CR1] Abdulai A, Aubert D (2004). Nonparametric and parametric analysis of calorie consumption in Tanzania. Food Policy.

[CR2] Akombi BJ, Agho KE, Merom D, Renzaho AM, Hall JJ (2017). Child malnutrition in sub-Saharan Africa: A meta-analysis of demographic and health surveys (2006–2016). PLoS ONE.

[CR3] Atack J, Bateman F, Haines M, Margo RA (2009). Did railroads induce or follow economic growth Urbanization and population growth in the American Midwest, 1850-60 (No. w14640). National Bureau of Economic Research.

[CR4] Basu S, Seligman H, Bhattacharya J (2013). Nutritional policy changes in the supplemental nutrition assistance program: A microsimulation and cost-effectiveness analysis. Medical Decision Making.

[CR5] Basu S, Yudkin JS, Berkowitz SA, Jawad M, Millett C (2018). Reducing chronic disease through changes in food aid: A microsimulation of nutrition and cardiometabolic disease among Palestinian refugees in the Middle East. PLoS Medicine.

[CR6] Benin, S., Thurlow, J., Diao, X., Kebba, A., & Ofwono, N. (2008). Agricultural growth and investment options for poverty reduction in Uganda. Discussion Paper 00790. Washington DC: International Food Policy Research Institute (IFPRI). Retrieved from http://www.ifpri.org/publication/agricultural-growth-and-investment-options-poverty-reduction-uganda

[CR7] Beyene, L., Namara, R., Sahoo, A., Shiferaw, B., Maisonnave, H., & Saltiel, G. (2018). Economywide and Distributional Impacts of Water Resources Development in the Coast Region of Kenya: Implications for Water Policy and Operations. World Bank, Washington, DC. World Bank. Retrieved from http://documents1.worldbank.org/curated/en/808491526362236466/pdf/126189-WP-P145559-PUBLIC-14-5-2018-12-9-47-W.pdf

[CR8] Blesh J, Hoey L, Jones AD, Friedmann H, Perfecto I (2019). Development pathways toward “zero hunger”. World Development.

[CR9] Boulanger, P., Dudu, H., Ferrari, E., Mainar-Causapé, A., Balié, J., & Battaglia, L. (2018). Policy options to support the Agriculture Sector Growth and Transformation Strategy in Kenya. A CGE Analysis, EUR 29231, Publications Office of the European Union, Luxembourg, 10.2760/091326.

[CR10] Boulanger, P., Dudu, H., Ferrari, E., Mainar-Causapé, A., & Ramos, M.P. (2020). Effectiveness of fertilizer policy reforms to enhance food security in Kenya: a macro–microsimulation analysis. Applied Economics, (first available online 02 sept). 10.1080/00036846.2020.1808180.

[CR11] Byrd K, Williams A, Dentz HN, Kiprotich M, Rao G, Arnold CD, Stewart CP (2017). Differences in complementary feeding practices within the context of the wash benefits randomized, controlled trial of nutrition, water, sanitation, and hygiene interventions in rural Kenya. The FASEB Journal.

[CR12] Carletto C, Corral P, Guelfi A (2017). Agricultural commercialization and nutrition revisited: Empirical evidence from three African countries. Food Policy.

[CR13] Chege CG, Andersson CI, Qaim M (2015). Impacts of supermarkets on farm household nutrition in Kenya. World Development.

[CR14] Chi DL, Masterson EE, Carle AC, Mancl LA, Coldwell SE (2014). Socioeconomic status, food security, and dental caries in US children: Mediation analyses of data from the National Health and Nutrition Examination Survey, 2007–2008. American Journal of Public Health.

[CR15] Chiputwa B, Qaim M (2016). Sustainability standards, gender, and nutrition among smallholder farmers in Uganda. The Journal of Development Studies.

[CR16] Cockburn, J., Savard, L., & Tiberti, L. (2014). Macro-micro models. Emerald Group Publishing Limited.

[CR17] De Haen H, Klasen S, Qaim M (2011). What do we really know? Metrics for food insecurity and undernutrition. Food Policy.

[CR18] Deaton A (1989). Rice prices and income distribution in Thailand: A non-parametric analysis. The Economic Journal.

[CR19] Deaton A (1989). Household survey data and pricing policies in developing countries. The World Bank Economic Review.

[CR20] Deaton A (1997). The analysis of household surveys: A microeconometric approach to development policy. The World Bank.

[CR21] Dercon S, Gilligan DO, Hoddinott J, Woldehanna T (2009). The impact of agricultural extension and roads on poverty and consumption growth in fifteen Ethiopian villages. American Journal of Agricultural Economics.

[CR22] Desiere S, Hung Y, Verbeke W, D’Haese M (2018). Assessing current and future meat and fish consumption in Sub-Sahara Africa: Learnings from FAO Food Balance Sheets and LSMS household survey data. Global Food Security.

[CR23] Donaldson D, Hornbeck R (2016). Railroads and American economic growth: A “market access” approach. The Quarterly Journal of Economics.

[CR24] Donini LM, Dernini S, Lairon D, Serra-Majem L, Amiot M-J, Del Balzo V, Giusti A-M, Burlingame B, Belahsen R, Maiani G, Polito A, Turrini A, Intorre F, Trichopoulou A, Berry EM (2016). A consensus proposal for nutritional indicators to assess the sustainability of a healthy diet: the Mediterranean diet as a case study. Frontiers in Nutrition.

[CR25] Ecker O, Qaim M (2011). Analyzing nutritional impacts of policies: An empirical study for Malawi. World Development.

[CR26] Ecker O, Weinberger K, Qaim M (2010). Patterns and determinants of dietary micronutrient deficiencies in rural areas of East Africa. African Journal of Agricultural and Resource Economics.

[CR27] FAO, Ifad, UNICEF, WFP & WHO. The State of Food Security and Nutrition in the World (2021). Transforming food systems for food security, improved nutrition and affordable healthy diets for all. Rome, FAO..

[CR28] FAO & Government of Kenya (2018). Kenya Food Composition Tables. Rome & Nairobi: The Food and Agriculture Organization of the United Nations & The Ministry of Health, Republic of Kenya & The Ministry of Agriculture and Irrigation, Republic of Kenya. http://www.fao.org/3/I8897EN/i8897en.pdf

[CR29] Food Security Information Network. (2019). Global Report on Food Crisis: Joint Analysis For Better Decision. Retrieved from https://www.fsinplatform.org/sites/default/files/resources/files/GRFC_2019-Full_Report.pdf

[CR30] Herforth A, Ballard TJ (2016). Nutrition indicators in agriculture projects: Current measurement, priorities, and gaps. Global Food Security.

[CR31] Hoddinott, J., & Yohannes, Y. (2002). Dietary diversity as a household food security indicator. Washington, D.C.: Food and Nutrition Technical Assistance Project, FHI 360, 2002.

[CR32] Development Initiatives. (2018). 2018 Global Nutrition Report: Shining a light to spur action on nutrition. Bristol, UK: Development Initiatives. Retrieved from https://globalnutritionreport.org/reports/global-nutrition-report-2018

[CR33] Kadiyala S, Harris J, Headey D, Yosef S, Gillespie S (2014). Agriculture and nutrition in India: Mapping evidence to pathways. Annals of the New York Academy of Sciences.

[CR34] Keats EC, Macharia W, Singh NS, Akseer N, Ravishankar N, Ngugi AK, Bhutta ZA (2018). Accelerating Kenya’s progress to 2030: Understanding the determinants of under-five mortality from 1990 to 2015. BMJ Global Health.

[CR35] Kennedy, G., Ballard, T., & Dop, M.C. (2010). Guidelines for Measuring Household and Individual Dietary Diversity. Rome: Nutrition and Consumer Protection Division, FAO. ISBN: 978-92-5-106749-9 Retrieved from http://www.fao.org/3/a-i1983e.pdf

[CR36] Kennedy GL, Pedro MR, Seghieri C, Nantel G, Brouwer I (2007). Dietary diversity score is a useful indicator of micronutrient intake in non-breast-feeding Filipino children. The Journal of Nutrition.

[CR37] Kenya Agricultural Research Institute. (2012). Food Security Report 2012. Retrieved from http://www.foodsecurityportal.org/kenya/food-security-report-prepared-kenya-agricultural-research-institute

[CR38] Kenya Ministry of Devolution and Planning. (2017). Implementation of the Agenda 2030 for Sustainable Development in Kenya, June 2017. Retrieved from https://www.un.int/kenya/sites/www.un.int/files/Kenya/vnr_report_for_kenya.pdf

[CR39] Kenya National Bureau of Statistics. (2018). Basic Report on the 2015/2016 Kenya Integrated Household Budget Survey. March. ISBN: 978-9966-102-03-4. Retrieved from https://sun-connect-news.org/fileadmin/DATEIEN/Dateien/New/KNBS_-_Basic_Report.pdf

[CR40] Kenya National Bureau of Statistics (2015). Kenya Demographic and Health Survey 2014. Nairobi, Kenya . Retrieved from http://dhsprogram.com/pubs/pdf/FR308/FR308.pdf.

[CR41] Key N, de Janvry A, Sadoulet E (2000). Transaction costs and agricultural household supply response. American Journal of Agricultural Economics.

[CR42] Koppmair S, Kassie M, Qaim M (2017). Farm production, market access and dietary diversity in Malawi. Public Health Nutrition.

[CR43] Mainar-Causapé A, Boulanger P, Dudu H, Ferrari E (2020). Policy impact assessment in developing countries using Social Accounting Matrices: The Kenya SAM 2014. Review of Development Economics.

[CR44] Mainar-Causapé, A., Boulanger, P., Dudu, H., Ferrari, E., McDonald, S., & Caivano, A. (2018). Social accounting matrix of Kenya 2014. EUR 29056 EN, Publications Office of the European Union, Luxembourg 10.2760/852198.

[CR45] Masset E, Haddad L, Cornelius A, Isaza-Castro J (2012). Effectiveness of agricultural interventions that aim to improve nutritional status of children: Systematic review. BMJ.

[CR46] McDonald, S. (2021). A Standard Applied General Equilibrium Model: Technical Documentation. STAGE_t Version: January 2021. Retrieved from http://cgemod.org.uk/STAGE_t%20Model%20tech.pdf

[CR47] McDonald, S., Thierfelder, K., & Aragie, E. (2016). STAGE_DEV Version 2: August 2016. A Static Applied General Equilibrium Model: Technical Documentation, mimeo. Retrieved from http://www.cgemod.org.uk/STAGE_DEV.pdf

[CR48] Minten B, Koru B, Stifel D (2013). The last mile(s) in modern input distribution: Pricing, profitability, and adoption. Agricultural Economics.

[CR49] Moltedo, A., Alvarez, C., Troubat, N., & Cafiero, C. (2018). Optimizing the use of ADePT-Food Security Module for Nutrient Analysis. ADePT-FSM Version 3. Rome: FAO Statistics Division (ESS), January. Retrieved from http://www.fao.org/fileadmin/templates/ess/foodsecurity/Optimizing_the_use_of_ADePT_FSM_for_nutrient_analysis.pdf

[CR50] Moltedo, A., Troubat, N., Lokshin, M., & Sajaia, Z. (2014). Analyzing food security using household survey data: streamlined analysis with ADePT software. The World Bank. Retrieved from http://documents.worldbank.org/curated/en/488671468168574663/Analyzing-food-security-using-household-survey-data-streamlined-analysis-with-ADePT-software

[CR51] Mozaffarian D, Liu J, Sy S, Huang Y, Rehm C, Lee Y, Micha R (2018). Cost-effectiveness of financial incentives and disincentives for improving food purchases and health through the US Supplemental Nutrition Assistance Program (SNAP): A microsimulation study. PLoS Medicine.

[CR52] Muthini D, Nzuma J, Qaim M (2020). Subsistence production, markets, and dietary diversity in the Kenyan small farm sector. Food Policy.

[CR53] Nechifor V, Ramos MP, Ferrari E, Laichena J, Kihiu E, Omanyo D, Kiriga B (2021). Food security and welfare changes under COVID-19 in Sub-Saharan Africa: Impacts and responses in Kenya. Global Food Security.

[CR54] Pandey VL, Dev SM, Jayachandran U (2016). Impact of agricultural interventions on the nutritional status in South Asia: A review. Food Policy.

[CR55] Pauw, K., Thurlow, J., & Ecker O. (2018). Micro-econometric and Micro-Macro Linked Models: Modeling Agricultural Growth and Nutrition Linkages: Lessons from Tanzania and Malawi. Development Policies and Policy Processes in Africa, edited by Henning, C., O. Badiane, and E. Krampe, 97–115. Cham: Springer. 10.1007/978-3-319-60714-6_5

[CR56] Qureshi ME, Dixon J, Wood M (2015). Public policies for improving food and nutrition security at different scales. Food Security.

[CR57] Rudolf R (2019). The impact of maize price shocks on household food security: Panel evidence from Tanzania. Food Policy.

[CR58] Ruel, M.T., Alderman, H., & the Maternal and Child Nutrition Study Group (2013). Nutrition-sensitive interventions and programmes: How can they help to accelerate progress in improving maternal and child nutrition? Lancet 2013. Maternal and Child Nutrition.

[CR59] Salois MJ, Tiffin R, Balcombe KG (2012). Impact of income on nutrient intakes: Implications for undernourishment and obesity. The Journal of Development Studies.

[CR60] Santeramo FG, Shabnam N (2015). The income-elasticity of calories, macro-and micro-nutrients: What is the literature telling us?. Food Research International.

[CR61] Integrated Food Security Phase Classification (IPC) Global Partners. (2012). IPC Technical Manual Version 2.0. Evidence and Standards for Better Food Security Decisions. Rome: The Food and Agriculture Organization of the United Nations. Retrieved from http://www.ipcinfo.org/fileadmin/user_upload/ipcinfo/docs/IPC-Manual-2-Interactive.pdf

[CR62] UNICEF & WHO. (2010). Indicators for assessing infant and Young child feeding practices. Part 2. Measurement. WHO library cataloguing-in publication data, 2010. Retrieved from https://apps.who.int/iris/bitstream/handle/10665/44306/9789241599290_eng.pdf

[CR63] Vigani M, Dudu H, Ferrari E, Mainar-Causapé A (2019). Estimation of food demand parameters in Kenya, EUR 29657 EN. Publications Office of the European Union, Luxembourg,.

[CR64] WHO (2003). Diet, nutrition and the prevention of chronic diseases: report of a joint WHO/FAO expert group. WHO Technical Report Series 916. Geneva: World Health Organization. 12768890

[CR65] WHO. (2010). Nutrition Landscape Information System (NLIS) country profile indicators: interpretation guide. Technical report, Geneva: World Health Organization. Retrieved from https://apps.who.int/iris/bitstream/handle/10665/332223/9789241516952-eng.pdf

[CR66] WHO. (2019). The WHO Child Growth Standards. Retrieved from http://www.who.int/childgrowth/en/

[CR67] Yu X, Shimokawa S (2016). Nutritional impacts of rising food prices in African countries: A review. Food Security.

[CR68] Zeller M, Diagne A, Mataya C (1998). Market access by smallholder farmers in Malawi: Implications for technology adoption, agricultural productivity and crop income. Agricultural Economics.

