# Supplementary material for: Do agri-food market incentives improve food security and nutrition indicators? a microsimulation evaluation for Kenya
Source: Food Secur. 2021 Sep 30;14(1):209–27. doi: 10.1007/s12571-021-01215-2 (PMC8483734; doi:10.1007/s12571-021-01215-2)
Supplement: Supplementary file 1 — Supplementary file1 (DOCX 91 kb) [file 12571_2021_1215_MOESM1_ESM.docx]

**Annex A: The CGE model and the Social Accounting Matrix**

XX-NA-XXXXX-EN-N

**A.1 The CGE model**

A CGE model provides the impacts on food price and food consumed quantities of a market access improvement scenario. For this purpose, we employed a version of the comparative static variant of the STatic Applied General Equilibrium model (STAGE) (McDonald, 2021) and its extension for the context of developing countries (STAGE-DEV) (McDonald et al., 2016). This annex provides a brief description of the model, database and simulation that provide the changes in food price and quantities consumed entering the microsimulation. Although the CGE plays a relevant part within the developed methodological approach, the main text focus on the most original part of it, i.e., the use of the household budget survey and food composition table data to provide impacts of policy shocks at household level. Interested readers can find the key feature of the CGE model, its database, and details of the scenario within this annex. More details on the model are provided in McDonald et al., (2016), on the SAM in Mainar-Causapé et al., (2018 and 2020) and on the scenario in Boulanger et al., (2020).

STAGE_DEV is a single-country CGE model which comprises a large number of economic sectors and households. The model follows the small open-economy assumption whereby domestic price changes do not impact world prices and an assumption of perfect competition i.e., prices and quantities are not subject to market power on the supply or demand side. The employed variant of STAGE-DEV accounts for the non-separability of the dual role of smallholders as producers and consumers. Subsistence farmers produce their Home Production for Home Consumption (HPHC), allocating production factors for their own consumption. Explicitly modelling household production, consumption and factor supply requires adjusting household factor supply and market clearing conditions and constraining the factor use in the own production activity through factor endowment. Smallholder producers are modelled as multiple-output producers with composition of output varying in response to changes in relative prices of commodities through a Constant Elasticity of Transformation (CET) function. Furthermore, the CGE model assumes household domestic migration within the country (driven by regional differences in incomes). The functional distribution of income changes as households migrate and transfer their respective factors (capital and labour). Importantly, only economic incentives are embodied within the behavioural assumption (McDonald et al., 2016). The model also adopts a flexible production function for agricultural activities, assuming imperfect substitution between intermediate inputs, labour, capital, and land composites (Constant Elasticity of Substitution (CES) function). Intermediate inputs (including seeds) display a perfect complementarity nesting using a Leontief production function. For seeds, at a lower-level nest, household-produced and commercial seeds (i.e., bought from market) are imperfect substitutes assuming a CES function nesting. Different labours (i.e.,, skilled, semiskilled, and unskilled) and capital (i.e., agricultural capital and livestock) are imperfect substitutes (CES nesting), allowing producers to switch to less expensive labour or more productive labour or capital types. The land composite allows imperfect substitution (CES function) between rain-fed land and a composite that combines irrigated land, water, and fertilisers as substitutes (CES function).

Macroeconomic closure rules allow for a realistic description of the Kenyan economy. Factors are fully employed, except the labour for which a constant rate of unemployment is assumed. The fixed supply of labour holds at national level while the regional supply is updated to reflect changes due to migration. Land is mobile across agricultural activities within each region. The exchange rate adjusts to keep the foreign savings at the base year level and avoid any additional creation of liabilities. Government savings are fixed, and government spending adjusts to accommodate change in government income. All changes are expressed in terms of the numéraire, which is the Producer Price Index (PPI).

**A.2 The Social Accounting Matrix**

A SAM for Kenya with base year 2014 (Mainar-Causapé et al., 2018) is estimated with specific accounts for the treatment of HPHC, and a regionalization based on agro-economic zoning and social characteristics. This matrix is consistent with latest national statistics and is estimated from national accounts and micro-data from the KIHBS 2005/06. Modelling HPHC involves expanding the structure of a SAM including extra commodities valued at basic prices (excluding margins and sale taxes) while marketed commodities are valued at purchaser/market prices (including margins and sale taxes). For this reason, the 2014 SAM for Kenya deviates from standard matrices. The classic Representative Household Groups (RHG), which gather household behaviour as consumers of goods and services and as providers of factors of production, show the behaviour of households as units of production of commodities. These accounts incorporate the economic behaviour of households as producers of food commodities (agricultural and livestock products) as well as cash crops. This requires separate accounts for commodities produced by these households for own consumption (HPHC as input or as a final product) and other marketed commodities (produced by households and by conventional productive activities). The Kenyan agricultural sector is split into six regions plus two largest metropolises, i.e., Nairobi and Mombasa. The considered regions (High rainfall, Semi-arid North, Semi-arid South, Coast, Arid North, and Arid South) reflect different agricultural production characteristics and cost structures. The spatial breakdown also applies to households as productive and institutional units. Households as institutions are disaggregated into rural and urban, according to the area of residence. Furthermore, in both Nairobi and Mombasa, households are disaggregated by quintiles of income.

**Annex B: Supplementary results: Carbohydrate and Fat effects – Market Access**

XX-NA-XXXXX-EN-N

Figure B1. Carbohydrate effect (% change in Daily Carbohydrates intakes per capita).

(a) Percentiles of expenditure per capita (b) Percentiles of DEC per capita

(c) HCE DDS (diet diversity indicator). (d) Min HAZ (stunting in children -5y)

Source: own micro-simulation results.

Figure B2. Fat effect (% change in Daily Fats intakes per capita).

(a) Percentiles of expenditure per capita (b) Percentiles of DEC per capita

(c) HCE DDS (diet diversity indicator). (d) Min HAZ (stunting in children -5y)

Source: own micro-simulation results.

**Annex C: Supplementary results: Sensitivity of Market Access shocks**

This annex reports the results of a sensitivity analysis on the market access shock. To perform this analysis, the central scenario where the reduction of trade and transportation margins fell by 30% is compared with two alternative scenarios, a more pessimistic (lower) and a more optimistic (upper), in which costs fall respectively by 15% and 45% (change in public investment remains the same in all scenarios). Average percentage change in prices and consumed quantities at the national level are presented in table C1 for the three alternative scenarios.

**Table C1.** Sensitivity Analysis – Improving Market Access scenarios national average (%) impacts over prices and consumed quantities of food groups.

|  | **Improving Market Access scenario** | | | | | |
| --- | --- | --- | --- | --- | --- | --- |
|  | *Average % changes at National level* | | | | | |
|  | *Prices* | | | *Consumed Quantities* | | |
|  | **lower** | **central** | **upper** | **lower** | **central** | **upper** |
| Beer | -0.137 | -0.278 | -0.424 | 0.086 | 0.182 | 0.288 |
| Bread and Cereals | -0.494 | -0.994 | -1.498 | 0.584 | 1.184 | 1.800 |
| Coffee, tea and cocoa | -0.137 | -0.278 | -0.424 | 0.088 | 0.185 | 0.291 |
| Fish and seafood | -0.008 | -0.024 | -0.048 | -0.165 | -0.315 | -0.450 |
| Food products n.e.c. Spices & Miscellaneous | 0.035 | 0.066 | 0.093 | -0.245 | -0.48 | -0.706 |
| Fruits | -0.392 | -0.79 | -1.193 | 0.639 | 1.293 | 1.963 |
| Meat | -0.065 | -0.134 | -0.207 | -0.051 | -0.098 | -0.139 |
| Milk, cheese and eggs | -0.163 | -0.332 | -0.508 | 0.143 | 0.301 | 0.473 |
| Mineral water, soft drinks, fruit and vegetable juices | -0.137 | -0.278 | -0.424 | 0.104 | 0.217 | 0.337 |
| Oils and fats | -0.429 | -0.862 | -1.302 | 0.621 | 1.258 | 1.909 |
| Roots and tubers | -0.437 | -0.879 | -1.325 | 0.788 | 1.592 | 2.413 |
| Spirits | -0.137 | -0.278 | -0.424 | 0.081 | 0.168 | 0.263 |
| Sugar, jam, honey, chocolate | -0.108 | -0.221 | -0.340 | 0.041 | 0.094 | 0.158 |
| Vegetables | -0.737 | -1.474 | -2.211 | 1.420 | 2.86 | 4.320 |
| Wine | -0.137 | -0.278 | -0.424 | 0.107 | 0.223 | 0.347 |

Source: own CGE model results. Note: Central Market Access scenario is the main scenario of 30% trade costs reduction, while the “lower” alternative simulates a cut of 15% and the “upper” a cut of 45% in trade costs.

The results show a linear behaviour of all FS&N impacts, indicating that the reduction of trade and transportation margin is crucial to quantify the final impacts of these policy on FS&N (Table C2). The presented scenarios provide an upper and a lower bound. These bounds do not affect nor the direction or the ranking of the results presented in the main text, following the central scenario.

**Table C2.** Sensitivity Analysis – Average (and *SD*) FS&N Impact (3 dimensions) of Improving Market Access.

|  | **Improving Market Access scenarios** | | |
| --- | --- | --- | --- |
|  | **lower** | **central** | **upper** |
| **Food Access (% change in food purchasing power)** | | | |
| National | 0.22 | 0.44 | 0.66 |
|  | *0.09* | *0.18* | *0.26* |
| Metropolis | 0.13 | 0.27 | 0.40 |
|  | *0.06* | *0.12* | *0.18* |
| Other urban | 0.19 | 0.37 | 0.56 |
|  | *0.08* | *0.17* | *0.25* |
| Rural | 0.24 | 0.49 | 0.74 |
|  | *0.08* | *0.16* | *0.25* |
| Stunting - Min HAZ <= -2 | 0.24 | 0.49 | 0.74 |
|  | *0.08* | *0.16* | *0.24* |
| **Food Sufficiency (% change in DEC per capita)** | | | |
| National | 0.52 | 1.06 | 1.62 |
|  | *0.30* | *0.61* | *0.92* |
| Metropolis | 0.27 | 0.56 | 0.86 |
|  | *0.07* | *0.15* | *0.24* |
| Other urban | 0.58 | 1.18 | 1.79 |
|  | *0.31* | *0.62* | *0.93* |
| Rural | 0.51 | 1.04 | 1.58 |
|  | *0.30* | *0.61* | *0.92* |
| Stunting - Min HAZ <= -2 | 0.50 | 1.01 | 1.54 |
|  | *0.29* | *0.59* | *0.88* |
| **Food Adequacy - Protein (% change in Daily Protein intakes per capita)** | | | |
| National | 0.51 | 1.04 | 1.58 |
|  | *0.32* | *0.63* | *0.96* |
| Metropolis | 0.25 | 0.51 | 0.79 |
|  | *0.07* | *0.15* | *0.23* |
| Other urban | 0.55 | 1.11 | 0.79 |
|  | *0.31* | *0.63* | *0.23* |
| Rural | 0.51 | 1.04 | 1.58 |
|  | *0.32* | *0.64* | *0.97* |
| Stunting - Min HAZ <= -2 | 0.50 | 1.02 | 1.56 |
|  | *0.31* | *0.63* | *0.95* |
| **Food Adequacy - Carbohydrate (% change in Daily Carbohydrate intakes per capita)** | | | |
| National | 0.55 | 1.11 | 1.69 |
|  | *0.33* | *0.67* | *1.01* |
| Metropolis | 0.28 | 0.59 | 0.90 |
|  | *0.09* | *0.18* | *0.29* |
| Other urban | 0.61 | 1.24 | 1.02 |
|  | *0.33* | *0.67* | *1.88* |
| Rural | 0.53 | 1.08 | 1.64 |
|  | *0.33* | *0.67* | *1.00* |
| Stunting - Min HAZ <= -2 | 0.51 | 1.04 | 1.56 |
|  | *0.31* | *0.62* | *0.95* |
| **Food Adequacy - Fat (% change in Daily Fat intakes per capita)** | | | |
| National | 0.46 | 0.94 | 1.43 |
|  | *0.25* | *0.50* | *0.75* |
| Metropolis | 0.25 | 0.51 | 0.79 |
|  | *0.06* | *0.13* | *0.20* |
| Other urban | 0.52 | 1.05 | 1.60 |
|  | *0.27* | *0.54* | *0.81* |
| Rural | 0.45 | 0.91 | 1.39 |
|  | *0.23* | *0.47* | *0.71* |
| Stunting - Min HAZ <= -2 | 0.45 | 0.92 | 1.40 |
|  | *0.25* | *0.50* | *0.75* |

Source: own micro-simulation results. Note: Central Market Access scenario is the main scenario of 30% trade costs reduction, while the “lower” alternative simulates a cut of 15% and the “upper” a cut of 45% in trade costs.
